# Sleep problems and sociodemographic characteristics of the COVID-19 survivors

**DOI:** 10.1192/j.eurpsy.2025.1265

**Published:** 2025-08-26

**Authors:** M. Turki, N. Bouattour, H. Ben Ayed, R. Jbir, W. Abid, S. Msaad, S. Kammoun, N. Halouani, S. Ellouze, J. Aloulou

**Affiliations:** 1 Psychiatry B; 2 Preventive medicine and hospital hygiene; 3 Pneumology, Hedi Chaker university hospital, Sfax, Tunisia

## Abstract

**Introduction:**

The COVID_19 pandemic has affected all facets of living, notably our sleep-wake cycle. In fact, sleep disturbances have been reported, not only during the acute phase, but also after the episode. Previous studies have shown that some socio-demographic characteristics are associated with sleep problems.

**Objectives:**

To assess the relationship between sleep disturbances and the sociodemographic profile of the COVID-19 survivors.

**Methods:**

We conducted a prospective cohort study including 121 Tunisian COVID-19 inpatients who had been discharged alive from hospital. Each enrolled patient was asked about the period before the hospital stay, and the 6-9 month-period after hospital discharge, using the Arabic validated version of Pittsburgh sleep quality index “PSQI” scale to assess sleep problems.

**Results:**

The median age of our participants was 59 years old. Among them, 62 (51.2 %) were females. One hundred and eleven patients were married (91.7%), 102 patients (84.2%) had at least a primary educational level, while 19 (15.7%) patients were illiterate. Among the participants 86 (71.07%) had a job at the time of the infection, among them, 9 (7.4%) were Heath care providers. Ninety-five (78.5%) participants were non-smokers. As for alcohol use, 11 patients (9.1%) consumed alcohol. According to PSQI, the incidence of sleep disturbances after COVID was 48.8%. Sleep duration, sleep quality as well as sleep onset latency, sleep disturbances and sleep efficiency were the most affected domains in the PSQI. Females showed significantly higher PSQI after the COVID-19 infection (p<0.001). Non-married participants reported significantly higher scores of hypnotic drug use and daytime dysfunction (p=0.006, p=0.02 respectively). Quality of sleep was significantly poorer in illiterate patients (p=0.036). Health care providers had worse daytime dysfunction in comparison to other occupations (p=0.035). Non-smokers showed a deteriorated sleep efficiency as well as daytime dysfunction (p=0.03, p=0.021 respectively). No statistical association was found between sleep problems and age nor alcohol consumption.

**Conclusions:**

Our study highlighted several associations between sleep problems and sociodemographic characteristics of COVID-19 survivors. This helps clinicians to pinpoint the at-risk people, in order to intervene when needed.

**Disclosure of Interest:**

None Declared

